# Persistent random deformation model of cells crawling on a gel surface

**DOI:** 10.1038/s41598-018-23540-x

**Published:** 2018-03-26

**Authors:** Hiroyuki Ebata, Aki Yamamoto, Yukie Tsuji, Saori Sasaki, Kousuke Moriyama, Thasaneeya Kuboki, Satoru Kidoaki

**Affiliations:** Laboratory of Biomedical and Biophysical Chemistry, Institute for Materials Chemistry and Engineering, Kyushu University, CE41-204, 744 Motooka, Nishi-ku, Fukuoka, 819-0395 Japan

## Abstract

In general, cells move on a substrate through extension and contraction of the cell body. Though cell movement should be explained by taking into account the effect of such shape fluctuations, past approaches to formulate cell-crawling have not sufficiently quantified the relationship between cell movement (velocity and trajectory) and shape fluctuations based on experimental data regarding actual shaping dynamics. To clarify this relationship, we experimentally characterized cell-crawling in terms of shape fluctuations, especially extension and contraction, by using an elasticity-tunable gel substrate to modulate cell shape. As a result, an amoeboid swimmer-like relation was found to arise between the cell velocity and cell-shape dynamics. To formulate this experimentally-obtained relationship between cell movement and shaping dynamics, we established a persistent random deformation (PRD) model based on equations of a deformable self-propelled particle adopting an amoeboid swimmer-like velocity-shape relationship. The PRD model successfully explains the statistical properties of velocity, trajectory and shaping dynamics of the cells including back-and-forth motion, because the velocity equation exhibits time-reverse symmetry, which is essentially different from previous models. We discuss the possible application of this model to classify the phenotype of cell migration based on the characteristic relation between movement and shaping dynamics.

## Introduction

Cell migration plays important roles in various physiological and pathological processes in living organisms such as embryogenesis, morphogenesis, immunological response^1^, wound healing^2^, cancer metastasis^3^, etc. The ability to characterize and predict the migration behaviors of various kinds of cells is an important issue not only from a biomedical viewpoint, but also from the perspective of basic science in molecular cell biology. In general, cells dynamically change their shape as a result of contraction by actomyosin and extension through protrusion of the plasma membrane driven by actin polymerization^4^. In a time-scale of from minutes to hours, an entire cell can move based on the sum of such local fluctuations in shape. For example, in the case of keratocytes, extension of the front part and retraction of the rear part occur simultaneously at a constant speed. As a result, the cell experiences ballistic motion with a constant shape^5^. In the case of Dictyostelium cells, local extension and contraction fluctuate spatiotemporally^6^. As a result, cell movement consists of an alternating series of directed motion and random turning, which is called persistent random motion^7^.

With regard to such persistent random motion, random walk-based models, such as the persistent random walk (PRW) model, have been proposed to reproduce the migration patterns, but only if the trajectory does not have strong spatiotemporal correlations^8–13^. However, the PRW model does not adequately explain ordered patterns of migration, such as rotation, oscillation, and zig-zag trajectories, because this model assumes Brownian motion. These ordered motions have been reported to derive from the spatiotemporal dynamics of pseudopodia^6,14–17^, i.e., cell-shape dynamics. Thus, to explain spatiotemporally correlated motion, we should consider the effect of the shaping dynamics. However, previous approaches to formulate cell-crawling have not adequately quantified the relationship between cell movement and shape fluctuations based on experimental data regarding actual shaping dynamics. Recently, as a model for the migration of keratocytes and Dictyostelium cells, a phenomenological cell-crawling model was proposed based on the assumption that cell velocity is determined by the cell shape^18^. However, such a shape-based formulation of movement has not been experimentally examined for persistent random motion.

In this study, we aimed to elucidate and formulate the relationship between movement and shape fluctuations through the quantitative analysis of cell-shaping dynamics. First, to clarify the quantitative relationship between velocity and shape, we experimentally characterized the crawling of fibroblast cells in terms of shape fluctuations, especially extension and contraction, by using an elasticity-tunable gel substrate to modulate cell shape. Through a Fourier-mode analysis of the shape, the cell velocity was found to follow the cell-shape dynamics, where the obtained velocity-shape relationship was equivalent to that of an amoeboid swimmer^19^. Next, to formulate such shape fluctuation-based cell movement, we proposed a persistent random deformation (PRD) model by incorporating the amoeboid swimmer-like velocity equation^19^ into model equations for a deformable self-propelled particle^18^. The PRD model fully explains the statistics and dynamics of not only movement but also cell shape, including the characteristic back-and-forth motion of fibroblasts. This reciprocating motion is due to the time-reverse symmetry of the amoeboid swimmer-like velocity equation^19^, which is essentially different from previous migration models. Through fitting of experimental data with the model, we quantitatively evaluated fitting parameters, such as mobility, relaxation time of shaping, and magnitude of the internal force. The dependence of the fitting parameters on elasticity revealed that cells showed strong adhesion and large internal force on stiffer gels, as previously reported^20^. Finally, we discuss the possible application of this PRD model to classify the phenotype of the migration of different kinds of cells based on their characteristic relations between movement and shaping dynamics.

## Results

### Movement and deformation of cells on a gel surface

First, to elucidate the phenomenological relationship between cell movement and deformation, we studied the movement and deformation of NIH 3T3-fibroblast cells on a hydrogel surface with different degrees of stiffness. Photocurable styrenated gelatin (StG) was used because the elasticity of the gel can be adjusted from 1 to 1000 kPa by changing the duration of light irradiation in a photocrosslinking procedure^21^ (see Method). To determine the cell response to the elasticity of the substrate, we used 35, 120, and 410 kPa gels. Because cell movement is restricted to the flat gel surface, we analyzed the shape and trajectory of the geometric center of the cell shape projected on an *x*-*y* plane. In this paper, we define cell movement as the translocation of a cell body from one site to another. The word “cell migration” is used when we focus on the properties of movement at a cell-population level.

**Figure 1:**
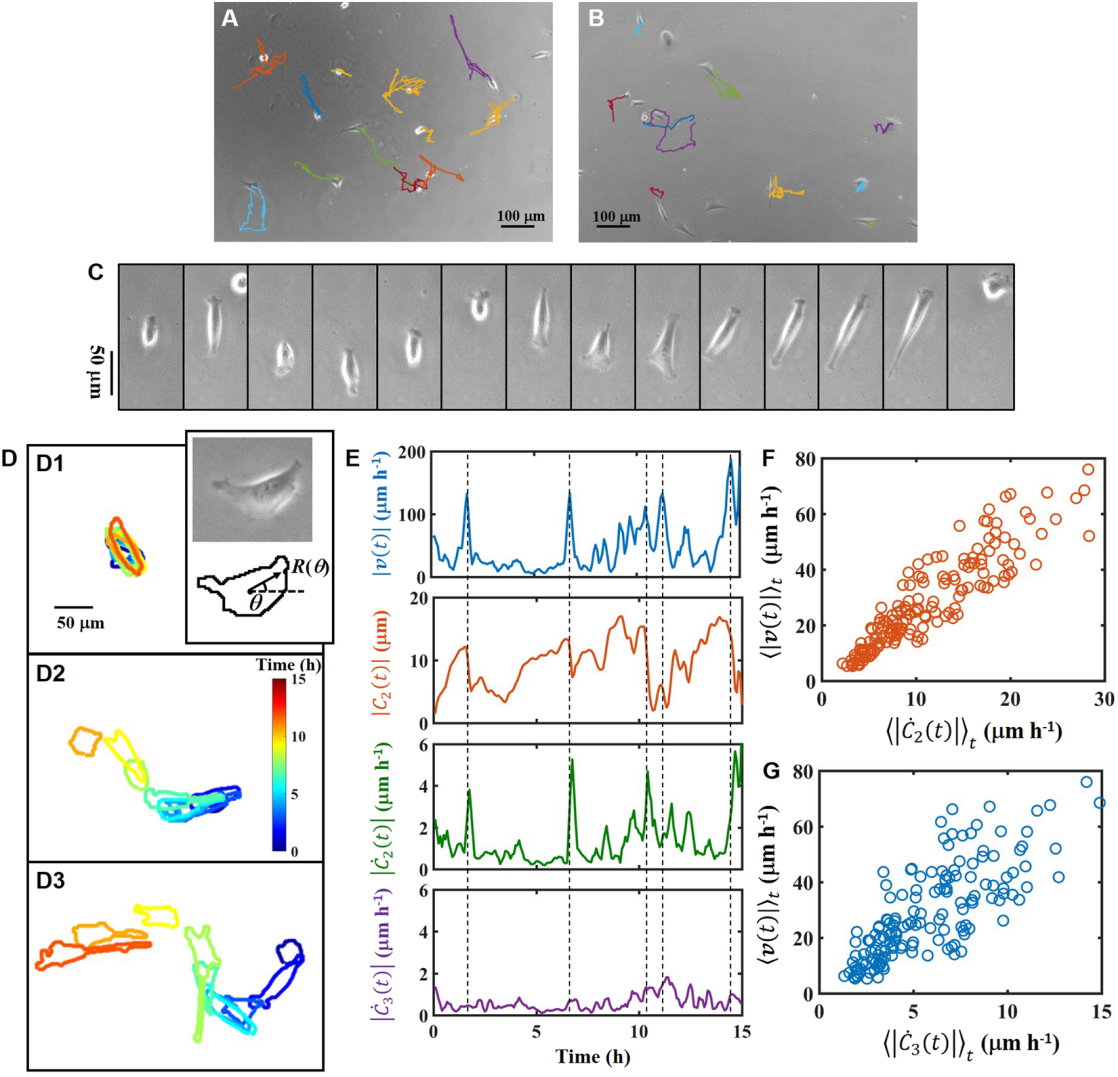
Cell movement with extension and contraction of the cell body. (**A**,**B**) Examples of the trajectories of cells on (**A**) 35 kPa and (**B**) 410 kPa gels. (**C**) Image sequence of a reciprocating cell. The time interval for each image is 25 min. (**D**) An example of the cell-shape dynamics. Color indicates the observation time (see color bar). Cells have average velocities of (D1) 10–20 μm/h, (D2) 20–30 μm/h, and (D3) 40–50 μm/h. Inset: Phase-contrast image of a cell and periphery of the cell detected by image analysis. (**E**) Time series of the magnitude of velocity |***v***(t)|, elongation |*C*_2_(*t*)|, time derivative of elongation $|{\dot{C}}_{2}|$, and time derivative of triangular deformation $|{\dot{C}}_{3}|$. Dashed lines indicate the peak positions of velocity. (**F**,**G**) Relation between the magnitude of velocity |***v***| and the time derivative of elongation $|{\dot{C}}_{2}|$ and triangular deformation $|{\dot{C}}_{3}|$. A symbol represents the time average of a cell. (**A**,**C**,**D**,**E**,**F**,**G**) Elasticity of the gel: 34 ± 18 kPa. (**B**) Elasticity of the gel: 412 ± 69 kPa. (**A**) N = 12. (**B**) N = 10. (**F**,**G**) N = 155.

Figure 1A,B show phase-contrast images of cell trajectories on 35 and 410 kPa gels, respectively. As the elasticity of the gel increases, the motility and persistence of motion decrease, and the cell body extends^20,22,23^. In all cases, the cells tend to show back-and-forth motion (Fig. 1C). Figure 1D shows time evolutions of movement and cell shape for cells on 35 kPa gel: Fig. 1D1–D3 show slow, middle, and fast cells, respectively. In all cases, the cells migrate along the long-axis of elongation^24,25^. When the cells change their direction of movement, they contract pseudopodia and then extend new ones. Thus, at the turning point of cell movement, the cells change their shape. As long as the cells remain elongated, they persistently migrate. A comparison of the slow and fast cells (Fig. 1D1,D3) shows that the fast cell repeatedly extends and contracts during migration. On the other hand, cells that maintain constant elongation do not move fast (Fig. 1D1).

Figure 1D implies that cell movement is correlated with the cell-shape dynamics. Here, we seek to quantify the relation between cell movement and shape. To evaluate cell shape quantitatively, we calculated the complex Fourier coefficient *C*_*n*_ of cell-shape *R* (see Method). The inset in Fig. 1D shows a phase-contrast image of a cell and its periphery detected by image analysis. The distance *R*(*θ*) from the centroid to the rim is calculated as a function of the angle *θ*, where *θ* is measured from the *x* axis (Fig. 1D, Inset). We averaged *R* over *θ* ± 6° for smoothing. Next, complex Fourier coefficient *C*_*n*_(*t*) is calculated from *R*(*θ*, *t*). Here, we focus on the elongation mode *C*_2_(*t*) and triangular mode *C*_3_(*t*). Figure 1E shows the time series of the magnitude of cell velocity |***v***(t)|, elongation |*C*_2_(*t*)|, time derivative of elongation $|{\dot{C}}_{2}|$, and time derivative of triangular deformation $|{\dot{C}}_{3}|$. |***v***(t)| tends to be large when elongation |*C*_2_(*t*)| varies rapidly. Thus, the time series of $|{\dot{C}}_{2}|$ closely resembles that of |***v***|. |***v***| is also moderately correlated with $|{\dot{C}}_{3}|$. This result indicates that a cell moves when the cell body extends or contracts. Figure 1F shows the relation between |***v***| and $|{\dot{C}}_{2}|$. Each symbol denotes the time average data of a cell. As expected, there is a strong positive correlation between these values (correlation coefficient *r* is 0.90). |***v***| is also positively correlated with $|{\dot{C}}_{3}|$ (*r* = 0.77, Fig. 1G). These results indicate that cells which exhibit frequent extension and contraction migrate faster. In a previous cell-crawling model^18^, |***v***| is conjectured to be proportional to |*C*_2_| and |*C*_3_|. However, Fig. 1D–G indicate that velocity ***v*** is positively correlated with ${\dot{C}}_{2}$ and ${\dot{C}}_{3}$, instead of *C*_2_ and *C*_3_.

### Migration law based on deformation

**Figure 2:**
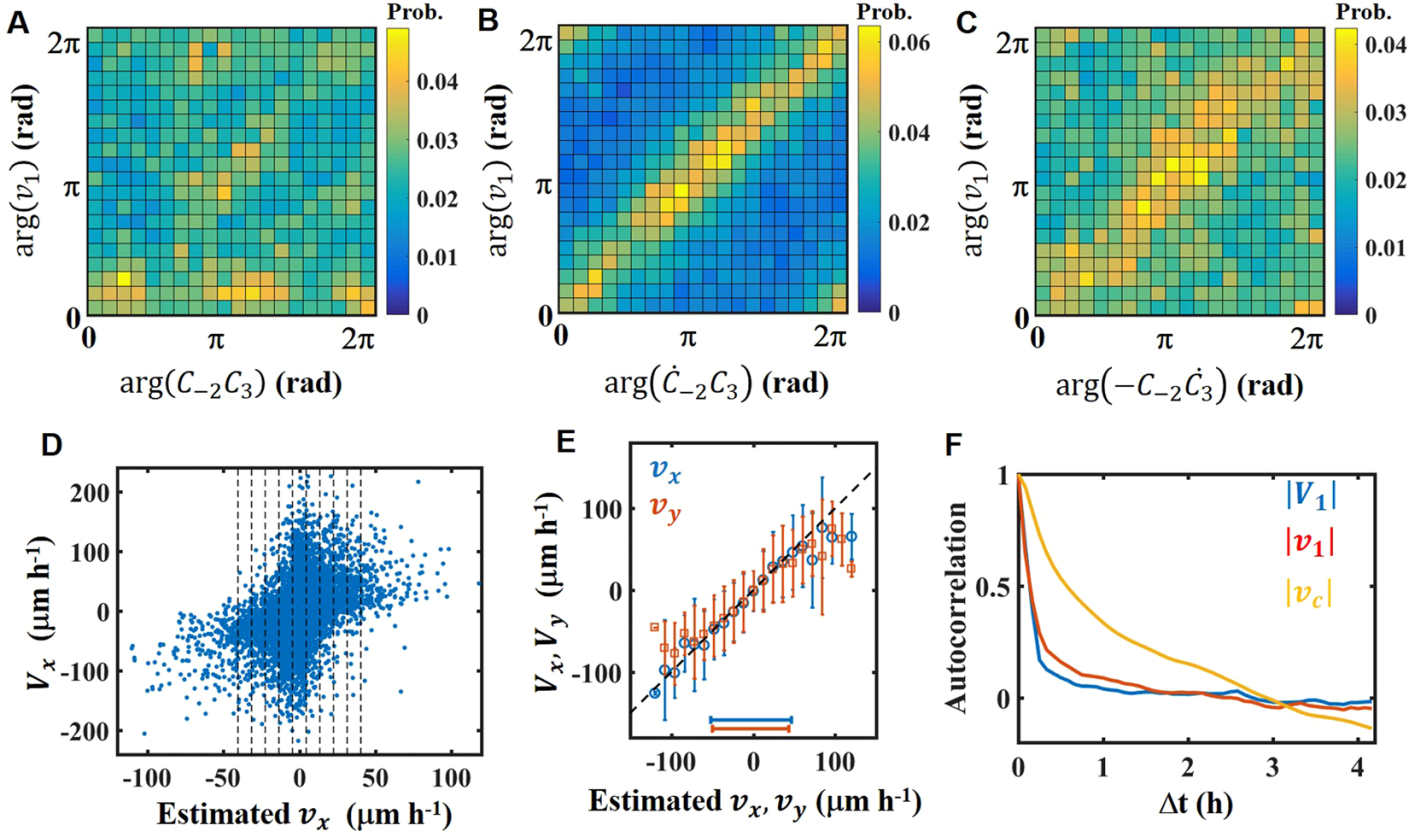
Robust relations among cell velocity and deformations. (**A**) Joint probability distribution between arg(*v*_1_) and $\text{arg}({C}_{-2}{C}_{3})$. (**B**) Joint probability distribution between arg(*v*_1_) and $\text{arg}({\dot{C}}_{-2}{C}_{3})$. (**C**) Joint probability distribution between arg(*v*_1_) and $\text{arg}({C}_{-2}{\dot{C}}_{3})$. (A to C) Yellow color indicates a high probability. (**D**) Scatterplot of the velocity *v*_*x*_ predicted from the model and the actual velocity *V*_*x*_. (**E**) Relation between *v*_*x*_ (*v*_*y*_), and *V*_*x*_ (*V*_*y*_). Black dashed line represents *v*_*x*_ = *V*_*x*_ (*v*_*y*_ = *V*_*y*_). Error bar indicates the standard deviation. Blue and red bars at the lower side of the figures denote the region where 99% of the data points are found. (**F**) Autocorrelation function of the velocity $|{v}_{1}|$ predicted from the model, actual velocity $|{V}_{1}|$, and velocity $|{V}_{c}|$ predicted from the previous model. (**A**,**F**) Elasticity of the gel: 34 ± 18 kPa. N = 155.

The results in the previous section suggest that cell movement is determined by the cell-shape dynamics. Since an equation that describes the relation between cell shape and velocity has not been elucidated for persistent random motion, we explored the velocity equation based on deformation, *C*_*n*_ and ${\dot{C}}_{n}$, experimentally. The previous cell-crawling model^18^ conjectured that velocity is determined by ${v}_{1}={\beta }_{c}{C}_{-2}{C}_{3}$, where ${v}_{1}={v}_{x}+i{v}_{y}$ and *C-*_2_ is a complex conjugate of *C*_2_. Here, we examine the velocity equation in terms of correlation among phases (arguments) of variables, *v*_1_, *C*_*n*_, and ${\dot{C}}_{n}$. The phases correspond to the direction of movement and deformation (see Method). When the previous model ${v}_{1}={\beta }_{c}{C}_{-2}{C}_{3}$ holds, $\text{arg}({v}_{1})=\text{arg}({C}_{-2}{C}_{3})$ must be satisfied for *β*_*c*_ > 0, where arg(*x*) is an argument of complex variable *x*. Figure 2A shows the joint probability distribution of arg(*v*_1_) and $\text{arg}({C}_{-2}{C}_{3})$. As shown, arg(*v*_1_) is not correlated with arg(*C*_−*2*_*C*_3_). Thus, ${v}_{1}={\beta }_{c}{C}_{-2}{C}_{3}$ does not hold for our experiment. Instead, we found that arg(*v*_1_) and $\text{arg}({\dot{C}}_{-2}{C}_{3})$ are closely correlated. As shown in Fig. 2B, the joint probability distribution of arg(*v*_1_) and $\text{arg}({\dot{C}}_{-2}{C}_{3})$ has a region of high probability around the diagonal line. This result indicates that $\text{arg}({v}_{1})=\text{arg}({\dot{C}}_{-2}{C}_{3})$. By analogy to the previous model, $\text{arg}({v}_{1})=\text{arg}({\dot{C}}_{-2}{C}_{3})$ suggests the relation ${v}_{1}={\beta }_{1}{\dot{C}}_{-2}{C}_{3}$. The relation ${v}_{1}={\beta }_{1}{\dot{C}}_{-2}{C}_{3}$ also satisfies the requirement that |***v***| is positively correlated with $|{\dot{C}}_{2}|$ (Fig. 1E,F). Similarly, we found a moderate correlation between arg(*v*_1_) and ${\rm{\arg }}(-{C}_{-2}{\dot{C}}_{3})$ (Fig. 2C). Figure 2C suggests that the velocity equation also includes the term $-{\beta }_{2}{C}_{-2}{\dot{C}}_{3}$. However, the term ${\beta }_{2}{C}_{-2}{\dot{C}}_{3}$ should be less dominant than ${\beta }_{1}{\dot{C}}_{-2}{C}_{3}$. Based on the above consideration, we propose that the velocity equation is

1 $${v}_{1}={\beta }_{1}{\dot{C}}_{-2}{C}_{3}-{\beta }_{2}{C}_{-2}{\dot{C}}_{3},$$

where *β*_1_ and *β*_2_ are fitting parameters. As *β*_1_ and *β*_2_ increase, cell velocity increases. Thus, *β*_1_ and *β*_2_ represent the mobility of the cell. This equation appeared to be equivalent to the migration law for a swimming amoeboid in a 3D fluid^19^. In general, terms with a higher mode, ${\dot{C}}_{-n}{C}_{n+1}$ and ${C}_{-n}{\dot{C}}_{n+1}$ with n > 3, could appear in the velocity equation^19^. However, in our experiment, ${\dot{C}}_{-2}{C}_{3}$ and ${C}_{-2}{\dot{C}}_{3}$ are the largest modes, and higher modes make only a small contribution. Thus, for simplicity, we do not include higher modes in Eq. (1). The equations for higher mode, *C*_*n*_ (n > 3), are discussed in the Supplementary Information (SI).

Here, we examine Eq. (1) experimentally, in terms of the measured velocity and deformation. We calculated the mobility *β*_1_ and *β*_2_ by least-squares fitting of Eq. (1). Estimated *β*_1_ and *β*_2_ for the three gels are listed in Table 1. As expected, *β*_2_ is smaller than *β*_1_ in all cases. As the elasticity of the gels increases, the mobility decreases. This result suggests that the resistance to motion increases as the elasticity of the substrate increases. By using estimated *β*_1_ and *β*_2_, we can compare the velocity *v*_1_ predicted from the model to the actual velocity *V*_1_ = *V*_*x*_ + *iV*_*y*_ measured in the experiment. The scatter plot of *v*_*x*_ and *V*_*x*_ in Fig. 2D implies that there is a positive correlation with large fluctuation. The correlation coefficient *r* between *v*_*x*_ and *V*_*x*_ is 0.39. To clarify the detailed relation, we average *V*_*j*_ in sections, $|{v}_{j}-\gamma i|\le \gamma /2,\,i\in {\boldsymbol{Z}},\,j=x,y$, where the boundaries of the sections are shown as dashed lines in Fig. 2D with *γ* = 12 μm h^−1^. In Fig. 2E, the symbols and error bars indicate mean values and standard deviations of *V*_*x*_ and *V*_*y*_. Blue and red bars at the lower side of the figures denote the region where 99% of the data points are included. For both *V*_*x*_ and *V*_*y*_, the symbols are well aligned on the black line that represents *v*_*x*_ = *V*_*x*_ and *v*_*y*_ = *V*_*y*_. Next, autocorrelation functions of $|{v}_{1}|$ and $|{V}_{1}|$ were compared (Fig. 2F). For comparison, autocorrelation functions of the velocity $|{v}_{c}|$ in a previous cell-crawling model^18^ were also calculated, where ${v}_{c}={\beta }_{c}{C}_{-2}{C}_{3}$. While the autocorrelation functions of $|{v}_{1}|$ and $|{V}_{1}|$ are very close, that of $|{v}_{c}|$ differs significantly. As shown in Fig. 2D, it is still hard to precisely predict the instantaneous velocity. However, on average, Eq. (1) describes the velocity dynamics well (Fig. 2E,F). Similar results were obtained for fibroblasts on 120 kPa and 410 kPa gels (see SI).

**Table 1:** Parameter estimation: velocity equation. List of fitting parameters in Eq. (1). Designation represents the elasticity of the gels. The fitting parameters are estimated through least-squares fitting. The minimum and maximum values show the 95% confidence interval. N = 155 for 35 kPa gel. N = 95 for 120 kPa gel. N = 119 for 410 kPa gel.

Designation	*β*_1_ (μm^−1^)	*β*_2_ (μm^−1^)
35 kPa gel	$0.29\pm 0.01$	$0.11\pm 0.01$
120 kPa gel	$0.20\pm 0.01$	$0.06\pm 0.01$
410 kPa gel	$0.19\pm 0.01$	$0.04\pm 0.01$

**Figure 3:**
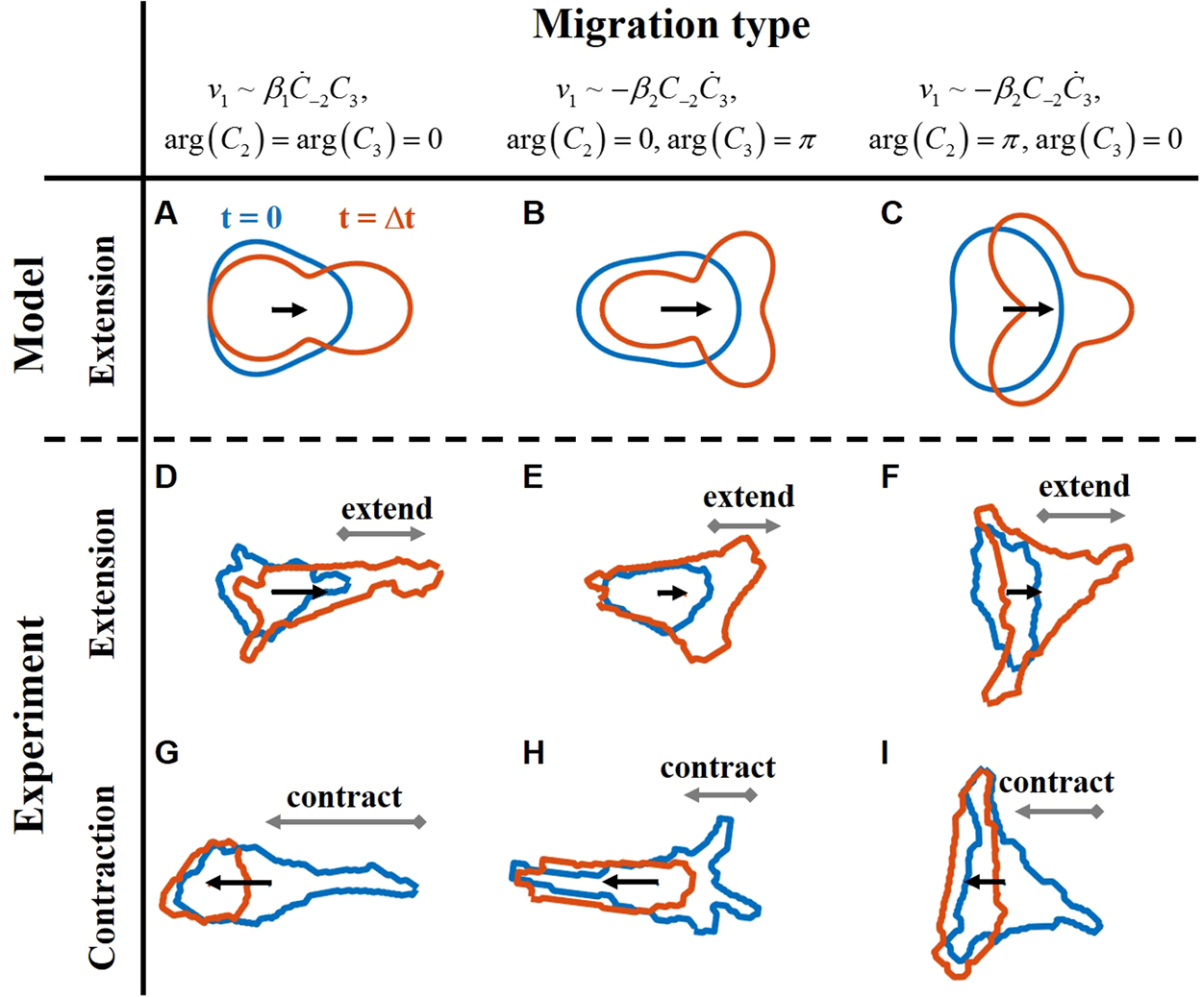
Examples of typical movement predicted from the velocity equation. Red curves show the initial cell shape. Blue curves shows the shape after some duration, Δ*t*. Arrows indicate the displacement of the centroid. (**A**,**C**) Cell movement calculated from Eq. (1). We assume linear increases of (A) $|{\dot{C}}_{2}|$ and (**B** and **C**) $|{\dot{C}}_{3}|$. (**A**) For the case in which elongation dominantly increases, $|{\dot{C}}_{2}|\gg |{\dot{C}}_{3}|$. $\text{arg}({C}_{2})=\text{arg}({C}_{3})=0$. (**B**,**C**) For the case in which triangular deformation dominantly increases, $|{\dot{C}}_{2}|\ll |{\dot{C}}_{3}|$. (**B**) $\text{arg}({C}_{2})=0,\,\text{arg}({C}_{3})=\pi$. (C) $\text{arg}({C}_{2})=\pi ,\,\text{arg}({C}_{3})=0$. (**D**–**F**) Extension processes of cells that correspond to (**A**–**C**). (**G** to **I**) Contraction processes of cells that correspond to reverse dynamics of (**A**–**C**). (**D** to **I**) Elasticity of the gel: 34 ± 18 kPa.

What can be predicted for cell movement based on Eq. (1)? Next, we discuss examples of the predicted dynamics in cell movement and deformation (Fig. 3A to C). Here, we consider the case of increasing |*C*_*n*_| with constant phases. In this case, a cell extends its body toward a certain direction. When the first term ${\beta }_{1}{\dot{C}}_{-2}{C}_{3}$ in Eq. (1) is dominant, arg(*C*_2_) = arg(*C*_3_) = 0 gives the dynamics that one of three pseudopodia extends (Fig. 3A). As a result, the cell body elongates and the centroid moves in the direction of the extended pseudopod. When the second term $-{\beta }_{2}{C}_{-2}{\dot{C}}_{3}$ in Eq. (1) is dominant, $\text{arg}({C}_{2})=0$ and $\text{arg}({C}_{3})=\pi$ give the dynamics for the extension of two pseudopodia (Fig. 3B). This process represents the extension of lamellipodia. In addition, $-{\beta }_{2}{C}_{-2}{\dot{C}}_{3}$ with $\text{arg}({C}_{2})=\pi$ and $\text{arg}({C}_{3})=0$ gives the case in which an elongated cell extends a new pseudopod (Fig. 3C). For the case of decreasing |*C*_*n*_|, movement with contraction is described. Since Eq. (1) exhibits time-reverse symmetry, contraction is described as the reverse of extension. Figure 3D to I show the corresponding dynamics observed in the experiment, including both extension and contraction. As expected from Eq. (1), it is hard to distinguish between extension and contraction based solely on a snapshot of the cell shape (Fig. 3D–I). However, there are differences in the cell-shape dynamics. For example, contraction is faster than extension. Consequently, we need to know the cell-shape dynamics to predict movement. This migration property is quite different from those in a previous cell-crawling model^18^ and keratocytes^5^, where velocity is determined by the instantaneous shape.

### Migration model and fitting of the experimental data

Equation (1) describes the dynamics of the velocity. However, we still don’t have the governing equations for *C*_2_ and *C*_3_ to describe the actual movement of a cell, i.e., the dynamics of the cell trajectory and shaping, because the velocity is determined by the time evolution of *C*_2_ and *C*_3_. Here, we propose a migration model by introducing time-evolution equations for *C*_2_ and *C*_3_ that are based on a previous cell-crawling model^18^. In the cell-crawling model, elongation and triangular deformations are induced by the force dipole *F*_2_ and force quadrupole *F*_3_ of the internal force acting on the periphery of the cell. We derive time-evolution equations for *C*_2_ and *C*_3_ by considering the force multipole and possible coupling terms (see Method):

2 $${\dot{C}}_{2}=-{\kappa }_{2}{C}_{2}-{\alpha }_{2}{v}_{-1}{C}_{3}+{F}_{2},$$

3 $${\dot{C}}_{3}=-{{{\rm K}}}_{3}{C}_{3}-{\alpha }_{3}{v}_{1}{C}_{2}+{\beta }_{3}{C}_{-3}{F}_{6}+{F}_{3},$$

4 $${{{\rm K}}}_{3}={\kappa }_{3}+{\gamma }_{3}{|{C}_{3}|}^{2}.$$

In these equations, *κ*_2_*C*_2_ and *K*_3_*C*_3_ cause relaxation to a circular shape, which corresponds to a restoring force. The terms *α*_2_*v*_−1_*C*_3_ and *α*_3_*v*_1_*C*_2_ represent changes in shape due to movement. As we explain later, *β*_3_*C*_−3_*F*_6_ is introduced to fit the long tail of the probability distribution function of *C*_3_. ${\gamma }_{3}|{C}_{3}{|}^{2}$ is a nonlinear damping term to suppress the divergence of *C*_3_ due to *β*_3_*C*_−3_*F*_6_. Similar to the noise term in the persistent random walk model^8–10^, we assume that the force multipole fluctuates randomly. Here, we use red noise with cut-off frequency *κ*_*f*_ for the force multipole, because the spatial distribution of the internal force may not change so frequently:

5 $${\dot{F}}_{i}={\kappa }_{f}(-{F}_{i}+{\sigma }_{i}{\xi }_{i}),$$

where *ξ*_*i*_ = *ξ*_*ix*_ + *iξ*_*iy*_. *ξ*_*ix*_ and *ξ*_*iy*_ are white Gaussian noise; $\langle {\xi }_{ij}\rangle =0,\,\langle {\xi }_{ij}{\xi }_{kl}\rangle ={\delta }_{ik}{\delta }_{jl}$, *i*, *k* = 2, 3, … and *j*, *l* = *x*, *y*. In the experiment, cells move along the long-axis of elongation (Fig. 1D). In our model, Eqs (1) to (5) are not sufficient to reproduce such movement, because Eq. (1) does not include a coupling term between *v*_1_ and *C*_2_. To resolve this discrepancy, we modify the velocity equation, Eq. (1), by adding a coupling term between *v*_1_ and *C*_2_:

6 $${{\Gamma }}_{v}{v}_{1}={\alpha }_{v}{v}_{-1}{C}_{2}+{\beta }_{1}{\dot{C}}_{-2}{C}_{3}-{\beta }_{2}{C}_{-2}{\dot{C}}_{3},$$

7 $${{\Gamma }}_{v}=1+{\gamma }_{v}{|{C}_{2}|}^{2}.$$

The additional term *α*_*v*_*v*_−1_*C*_2_ causes movement along the long-axis of elongation for *α*_*v*_ > 0^25,26^. In the previous model, velocity regulates the direction of elongation^18^. In our model, elongation regulates the direction of movement of the cell. We also add the nonlinear damping term ${\gamma }_{v}|{C}_{2}{|}^{2}$ to suppress the divergence of *v*_1_ due to *α*_*v*_*v*_−1_*C*_2_. We confirmed that Eq. (6) is a better model than Eq. (1) in terms of Akaike’s Information Criterion (AIC)^27^. For 35 kPa gel, Eq. (1) gives AIC = $5.352\times {10}^{4}$, and Eq. (6) gives AIC = $5.263\times {10}^{4}$ (see SI). Eventually, velocity *v*_1_ is fully determined by cell deformations *C*_2_ and *C*_3_ that are randomly activated by internal forces *F*_2_ and *F*_3_. Since *C*_2_ and *C*_3_ should have persistence through terms *κ*_2_*C*_2_ and *K*_3_*C*_3_, the migration model, Eqs (2) to (7), can be referred to as a persistent random deformation (PRD) model.

**Figure 4:**
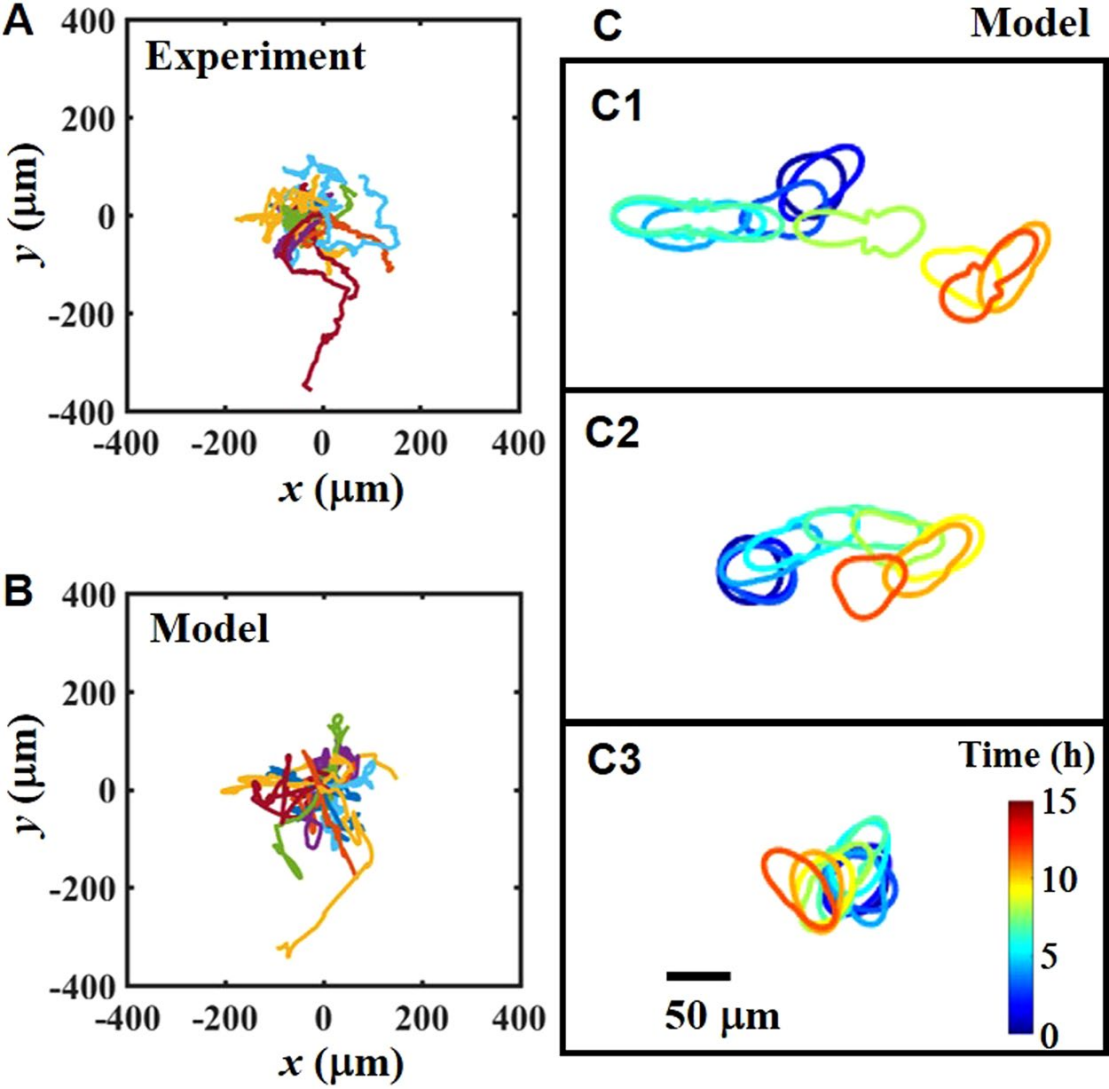
Cell trajectory and cell shape calculated from the PRD model. (**A**,**B**) Starting position-superimposed trajectories of the cells for (**A**) the experiment and (**B**) the numerical simulation. (**C**) Examples of the cell-shape dynamics obtained from the numerical simulation. Color indicates the observation time. Cells have an average velocity of (C1) 10–20 μm/h, (C2) 20–30 μm/h, and (C3) 40–50 μm/h. (**A**) Elasticity of the gel: 34 ± 18 kPa. N = 22. (**B**,**C**) We used fitted parameters for 35 kPa gels listed in the SI. To reconstruct the shape, we considered higher modes, n < 7 (See SI).

Figure 4A and B show the trajectories obtained by experiment and numerical simulation with Eqs (2) to (7) (see also SI and movies). Although the model does not have an inertia term in the velocity equation, the trajectory consists of persistent motion and rapid turning (Fig. 4B). Figure 4C show the cell-shape dynamics calculated in the simulation. In the simulation, we calculate *C*_4_, *C*_5_, and *C*_6_ (see SI). Since inertial forces *F*_2_ and *F*_3_ fluctuate, the cell shape repeatedly extends and contracts. At the turning point, cells change the direction of the deformation. Cells that experience frequent extension and contraction move faster (Fig. 4C). Thus, the PRD model well explains the cell movement and shaping in the experiment (Fig. 1D). In the PRD model, the cell-shape dynamics and force gradually follow the fluctuation due to the time-retardation term, 1/*κ*_*i*_. As a result, elongation and triangular deformation persist for a few hours. Since the direction of movement is approximately parallel to the direction of elongation (Fig. 4C), persistent random motion in the PRD model arises from persistent fluctuation of the cell shape, as we expected.

**Figure 5:**
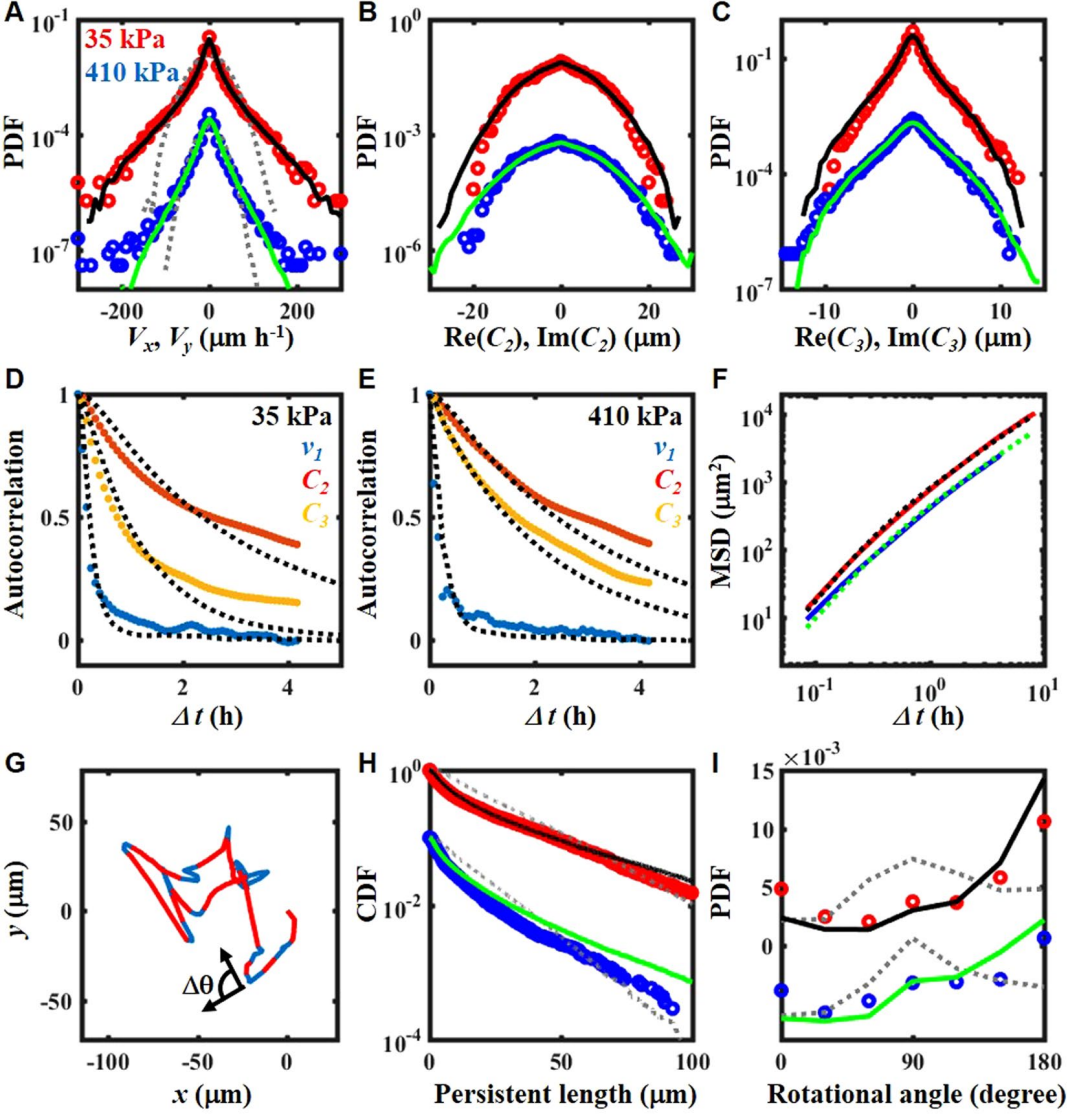
Properties of the cell velocity, deformation and trajectory. (**A**,**C**) Probability distribution function (PDF) of (**A**) velocity, (**B**) elongation, and (**C**) triangular deformation. (**D**,**E**) Autocorrelation function of the cells on (**D**) 35 kPa and (**E**) 410 kPa gels. Red: elongation. Yellow: triangular deformation. Blue: velocity. Black dashed lines: fitted curve. (**F**) Mean square displacement (MSD). Red: 35 kPa gel. Blue: 410 kPa gel. Black: fitting curve for 35 kPa gel. Green: fitting curve for 410 kPa gel (**G**) An example of the cell trajectory. The trajectory was drawn with red lines when cells moved persistently. An example of a rotation angle *Δθ* is illustrated. (**H**) Complementary cumulative distributions (CDF) of the persistent length. (**I**) PDF of the rotation angle. (A to C, H, and I) Red circle: 35 kPa gel. Blue circle: 410 kPa gels. Black line: fitting curve for 35 kPa gel. Green line: fitting curve for 410 kPa gel. (**A**,**H**,**I**) Gray dashed lines: fitted curves by the PRW model. N = 155 for 35 kPa gel. N = 119 for 410 kPa gel. (**A** to **C**,**H**,**I**) For clarity, symbols for the 410 kPa gel are shifted (A to C) 1/100, (H) 1/10, and (I) −0.005.

The detailed properties of migration dynamics are shown in Fig. 5, along with a comparison of the experimental and simulated results. In this figure, red and blue symbols represent the data obtained on 35 and 410 kPa gels, respectively. For clarity, symbols for the 410 kPa gel are shifted downward. Black and green lines are fitting curves for Eqs (2) to (7) for cells on 35 and 410 kPa gels, respectively (see Method). Note that the statistical properties of the results in Fig. 4 are shown as data for the 35 kPa gel in Fig. 5. The experimental and simulated data for 120 kPa gel are shown in the SI. The properties of migration and deformation are well-fitted by the model. For comparison, we also fitted the same experimental data by the conventional PRW model (gray lines in Fig. 5A,H,I, see Method). The PRW model reproduces the MSD and autocorrelation function of velocity (see SI). However, the model cannot explain the PDFs of velocity, persistent length, or rotation angle (Fig. 5A,H,I). Fitting by the PRW model is described in detail in the SI.

### Statistical properties of cell velocity, deformation and trajectory

We evaluated the statistical properties of *v*_1_, *C*_2_, and *C*_3_ through a probability distribution function (PDF) for the migration of a cell population. Due to spatial symmetry, the real and imaginary parts of *v*_1_, *C*_2_, and *C*_3_ obey the same distribution function. Thus, in Fig. 5A,C, we averaged the PDFs of the real and imaginary parts of the variables. The PDF of the velocity has a non-Gaussian tail (Fig. 5A). We found that the PDF of velocity approximately obeys an exponential distribution, as previously reported^28,29^. In the PRD model, the term *α*_*v*_*v*_−*1*_*C*_2_ in Eq. (6) causes the exponential tail of the velocity distribution (Fig. 5A). Without the nonlinear damping term, ${\gamma }_{v}|{C}_{2}{|}^{2}$ in Eq. (7), the velocity would show a power law distribution^30,31^. The tail of the PDF of elongation resembles a Gaussian distribution, but the peak of the PDF is sharper than that of a Gaussian distribution (Fig. 5B). The PDF of triangular deformation has an exponential tail (Fig. 5C). In the PRD model, the term *β*_3_*C*_−3_*F*_6_ in Eq. (3) gives the exponential tail of the PDF of triangular deformation (Fig. 5C), because the term behaves as multiplicative noise^30,31^. In addition to the PDFs of *v*_1_, *C*_2_, and *C*_3_, those of phase differences among velocity and deformations are also well fitted (see SI).

Figure 5D,E show autocorrelation functions of *v*_1_, *C*_2_, and *C*_3_ for 35 and 410 kPa gels. The dashed lines represent fitting curves by the model. The relaxation times of *C*_2_ and *C*_3_ are long compared to *v*_1_, which denotes a long persistence of the cell shape. The non-Gaussian distribution of *C*_2_ and *C*_3_ (Fig. 5B,C) and the long persistence (Fig. 5D,E) indicate the persistent non-Gaussian fluctuation of cell deformation. Because the cells migrate in the direction of elongation (Fig. 1D) and the relaxation of elongation is slow (Fig. 5D,E), the trajectories of the cells tend to follow a straight line.

Figure 5F shows the mean square displacement (MSD) of the cells. Similar to previous results^13,28,32^, the exponents of MSD were 1.6–1.7 at a short time interval. At a long time interval, the exponents came close to 1 and the dynamics became diffusive. The cell trajectory consisted of a series of persistent motion and rapid turning^7,13^. Thus, we investigated the persistence and turning angle of the trajectory (Fig. 5G), which are defined in the Method section. Figure 5H show the complementary cumulative distributions (CDF) of the persistent length. The CDF of the persistent length has an exponential tail, as reported previously^13,32^. The PDF of the rotation angle Δ*θ* has a large peak at 180° (Fig. 5I), which is quite different from previous studies with persistent random walk-based models^7,13^. The angle 180° represents reciprocal motion (Fig. 1C), but the back-and-forth motion is not periodic (Fig. 5D,E). The peak decreases as the elasticity of the substrate increases. Thus, back-and-forth motion is suppressed on the stiff gel. In contrast to PRW models that violate time-reverse symmetry, the time-reverse symmetry of the velocity equation, Eq. (6), causes this strong anti-correlation of turning angles.

### Dependence of the cell properties on the elasticity of the substrate

We now summarize the cell response to the elasticity of the substrate. Some of the fitting parameters for the three gels are shown in Table 2. All sets of fitting parameters are listed in SI. Table 2 shows that the mobility *β*_1_ and *β*_2_ significantly decreased as the elasticity of the substrate increased. This result suggests that, for a stiff substrate, cells strongly stick to the substrate^20^. The relation *κ*_2_ < *κ*_3_ indicates that elongation is the most unstable and ‘active’ Fourier mode. As the substrate become stiffer, *κ*_3_ significantly decreases while *κ*_2_ is constant. This implies that triangular deformation is enhanced on a stiff gel.

**Table 2:** Parameter estimation: Persistent random deformation model. List of fitting parameters in Eqs (2) to (7) used in Figs 3, 4 and 5. The method of fitting is described in Method. The designation represents the elasticity of the gels. Mean radius *R*_0_ is obtained from experimental data. *R*_0_ = 23.9, 26.6, and 27.2 μm for 35, 120, and 410 kPa gels, respectively.

Designation	*β*_1_ (μm^−1^)	*β*_2_ (μm^−1^)	*κ*_2_ (h^−1^)	*κ*_3_ (h^−1^)	*σ*_2_/(*R*_0_*κ*_2_)	*σ*_3_/(*R*_0_*κ*_3_)
35 kPa gel	1.21 ± 0.03	0.24 ± 0.01	0.38 ± 0.00	1.55 ± 0.05	0.66 ± 0.01	0.026 ± 0.001
120 kPa gel	0.83 ± 0.02	0.15 ± 0.01	0.40 ± 0.01	1.11 ± 0.02	0.64 ± 0.01	0.036 ± 0.001
410 kPa gel	0.59 ± 0.02	0.07 ± 0.00	0.40 ± 0.01	0.51 ± 0.02	0.67 ± 0.03	0.076 ± 0.003

Finally, we performed a dimensional analysis to estimate the magnitude of internal forces. In the PRD model, we assume that the coefficients of ${\dot{C}}_{2}$ and ${\dot{C}}_{3}$ are unity. As a result, the dimensions of *σ*_2_ and *σ*_3_ become μm h^−1^. Here, we consider *C*_*n*_/*R*_0_ to be the strain for the cell body, where *R*_0_ is the mean radius of the cell. We also define *E* as the elasticity of the cell. If we consider that the terms *κ*_2_*C*_2_ and *κ*_3_*C*_3_ in Eqs (2) and (3) cause relaxation to a circular shape, these terms should correspond to the restoring force *EC*_*n*_/*R*_0_. Thus, by multiplying Eqs (2) to (4) by *E*/(*R*_0_*κ*_*n*_), we can estimate the magnitude of the internal forces to be *Eσ*_*n*_/(*R*_0_*κ*_*n*_). In our analysis, we can calculate the non-dimensional forces, *σ*_*n*_/(*R*_0_*κ*_*n*_). Table 2 shows a constant value in the non-dimensional force dipole, *σ*_2_/(*R*_0_*κ*_2_), while the non-dimensional force quadrupole, *σ*_3_/(*R*_0_*κ*_3_), increases significantly on a stiffer substrate. It has been reported that the elasticity of fibroblast cells increases as the substrate becomes stiffer^23^. Eventually, internal forces should increase as the elasticity of the substrate increases^20^.

## Discussion

In this study, we applied a Fourier mode analysis of the cell shape to quantitatively examine the relation between movement and shaping dynamics. The Fourier mode analysis revealed that the velocity of fibroblast cells is a function of the time derivative of the elongation and triangular deformation of the cell. We sought to apply our analytical method to other cells. For example, keratocytes are well-known migratory cells^5^. Keratocytes migrate in a manner that is quite different from that of fibroblasts. Fibroblasts migrate along the long axis of the cell body through extension and contraction. On the other hand, keratocytes migrate along the short axis of the cell body while retaining an almost constant shape^5^. Equation (1) cannot explain the migration dynamics of keratocytes because Eq. (1) with a constant shape gives a motionless state. Thus, the previous model of cell-crawling is adequate for a migration model of keratocytes^18^, which assumes that the velocity is proportional to *C*_−*2*_*C*_3_. If we combine Eq. (1) with the previous model, the general form of the migration law can be written as

8 $${v}_{1}={\beta }_{1}{\dot{C}}_{-2}{C}_{3}-{\beta }_{1}{C}_{-2}{\dot{C}}_{3}+{\beta }_{c}{C}_{-2}{C}_{3},$$

where *β*_*c*_ = 0 gives a migration model of fibroblast-like cells and *β*_1_ = *β*_2_ = 0 gives a migration model of keratocytes. Dictyostelium cells seem to have intermediate properties. When we focus on the phases of velocity and deformation, arg(*v*_1_) is correlated with arg(*C*_−*2*_*C*_3_)^24^. This implies that the velocity equation includes the term *β*_*c*_*C*_−*2*_*C*_3_. However, in contrast to keratocytes, the shape of Dictyostelium cells varies considerably over time^6^. Thus, extension and contraction, Eq. (1), could also be included in the velocity equation. Although the above discussion is merely a conjecture, a systematic investigation of the relation between cell velocity and shape may be useful for classifying the migration type of cells.

In this work, we found that the movement of fibroblast cells on a gel surface is not explained by previous migration models. We quantitatively show the correlation between cell movement and extension/contraction of the cell body. We propose a persistent random deformation (PRD) model that is based on extension/contraction-based movement. The PRD model shows good agreement with the statistical properties of the trajectory, velocity and shape of the cell, including persistent non-Gaussian fluctuation of deformation. By fitting experimental data to the model, we quantitatively evaluate the coefficients in Eqs (2) to (7), such as motility parameters, the relaxation time of shaping, and the magnitude of internal force. With regard to a theoretical viewpoint, it is important to clarify the physical meaning of the coefficients by solving the continuous migration model^33,34^ based on the dynamics of focal adhesion and actin^35^. The movement and shaping of cells should be regulated by the dynamics of focal adhesion, stress fibers and an actin network^35^. Thus, with regard to an experiment, the simultaneous measurement of such internal structures could provide a better understanding of migration law Eq. (1) and the PRD model. Since the PRD model can be applied regardless of the details of the cell and its environment, we can evaluate the dependence of cell properties on the culture environment through estimation of the fitting parameters. In summary, the proposed model and analytical method provide a new tool for investigating the migration dynamics of cells.

## Methods

### Preparation of StG gel substrate

Photocurable styrenated gelatin (StG) was used as a culture substrate. The preparation method has been described previously^21,36,37^. StG (30 wt%) and sulfonyl camphorquinone (2.5 wt% of gelatin; Toronto Research Chemicals, ON, Canada) were dissolved in phosphate-buffered saline (PBS). The mixed solution was centrifuged and aspirated to exclude deposits and dissolved oxygen. The sol solution was then conditioned using an AR-100 deforming agitator. 30 μl of the StG sol solution was spread between vinyl-silanized glass substrates (vinyl-glass) and a normal glass substrate coated with poly(N-isopropylacrylamide) (PNIPAAm, Sigma Aldrich, St. Louis, MO). The gel was then prepared by irradiation of the entire sample with visible light (62 or 63 mW/cm^2^ at 488 nm; light source: MME-250; Moritex Saitama, Japan). Finally, the gels were detached from the PNIPAAm-coated normal glass substrate and washed thoroughly with PBS at 28 °C to remove adsorbed PNIPAAm. The elasticity of the gel was varied by changing the duration of irradiation from 300 s to 660 s. As we reported previously, the surface biochemical conditions were ensured to be the same for all the gel samples with different elasticities^21^. Therefore, the present experiments enabled us to investigate the pure biomechanical aspects of cell migration. The surface elasticity of the StG gel was determined by nano-indentation analysis using an atomic force microscope (JPK NanoWizard 4, JPK Instruments). A commercial silicon-nitride cantilever with a tetrahedral tip and a nominal spring constant of 0.1 N/m was used (BioLever mini, Olympus). We made three hydrogels with Young’s moduli of 34 ± 18 kPa, 121 ± 46 kPa, and 412 ± 69 kPa, respectively.

### Cell culture

NIH 3T3 fibroblast cells were cultured in Dulbecco’s Modified Eagle Medium (DMEM, Nacalai Tesque) at 37 °C in a humidified atmosphere containing 5% CO_2_.

### Time-lapse observation of cell migration

The migratory motion of cells on gels was monitored using an automated all-in-one microscope with a temperature- and humidity-controlled cell chamber (BIO REVO BZ-9000; Keyence Corporation, Osaka, Japan). Prior to the time-lapse observations, cells were seeded onto the gel surface at a density 1.5 × 10^3^ cells/cm^2^ and cultured for 6 hours under 5% CO_2_. Phase-contrast images of cells were captured every 5 min for 15–20 h.

### Analysis of cell trajectories and cell shape

Movement trajectories and the shape of the cells were determined and analyzed using MATLAB software. Based on the edge detection of the cell, we extracted the shape of the cell from the phase-contrast images (Fig. 1D). The details of image-processing are explained in SI. We traced each cell and measured the time evolution of the trajectory and shape. If the cells collided or if a cell replicated, we stopped the trace. When the cells separated again, we renumbered the cells and restarted the trace. Thus, the cells have different durations of data. We only analyzed data that accumulated for longer than 8.3 h (35 kPa and 120 kPa gels) or 4.2 h (410 kPa gel). Through the image analysis, cell trajectories *x*(*t*) and shape *R*(*θ*, *t*) were calculated (Fig. 1D inset). The complex Fourier coefficient *C*_*n*_(*t*) of the spatiotemporal shape *R*(*θ*, *t*) is defined as

9 $$R(\theta ,t)={R}_{0}+\sum _{n=2}^{m}({C}_{n}(t){e}^{in\theta }+{C}_{-n}(t){e}^{-in\theta }),$$

where *R*_0_ is the mean radius and *m* is the number of data points. The mode n = 1, *C*_1_(*t*), almost always vanishes because *C*_1_(*t*) corresponds to displacement of the centroid. Thus, *C*_1_(*t*) is included in the velocity of the centroid^25,26^. The amplitude |*C*_*n*_(*t*)| corresponds to the magnitude of deformation, and the phase *ϕ*_*n*_ represents the direction of maximum deformation, where *ϕ*_*n*_ is defined as ${C}_{n}(t)=|{C}_{n}(t)|\exp in{\varphi }_{n}$. Before calculating velocity ${\boldsymbol{v}}(t)$ and ${\dot{C}}_{n}(t)$, we take the moving average of ${\boldsymbol{x}}(t)$ and *C*_*n*_(*t*) over 3 consecutive data points. *β*_1_ and *β*_2_ in Eq. (1) are estimated by minimizing the distance *S* between the velocity ${\boldsymbol{V}}(t)$ obtained experimentally and the velocity ${\boldsymbol{v}}(t)$ in the model; $S={\sum |{\boldsymbol{V}}-{\boldsymbol{v}}|}^{2}$. The solution of ${\partial }_{{\beta }_{1}}S={\partial }_{{\beta }_{2}}S=0$ gives *β*_1_ and *β*_2_.

### Coupling terms in the model

Based on the symmetry argument, we explain how coupling terms in the model are determined. A necessary condition for the coupling term is to satisfy the fundamental symmetries, uniformity and isotopy of the space^25,26,38^. The model equations should be invariant under a rotational transformation of coordinates. For simplicity, we define ${C}_{1}={v}_{1}$ and ${\varphi }_{1}={\varphi }_{v}$. When we rotate the cell *θ*_0_ radians, ${C}_{n}(t)=|{C}_{n}(t)|\exp in{\varphi }_{n}$ is transformed into $|{C}_{n}(t)|\exp in({\varphi }_{n}+{\theta }_{0})={C}_{n}\exp in{\theta }_{0}$. Similarly, the 2^nd^-order nonlinear term ${C}_{l}{C}_{m}$ is transformed into ${C}_{l}{C}_{m}\exp i(l+m){\theta }_{0}$. In the equation for *C*_*n*_, we require that *C*_*n*_ and ${C}_{l}{C}_{m}$ undergo the same transformation, otherwise the equation changes under rotational transformation. Thus, *n* = *l* + *m* is required. For example, *C*_*−1*_*C*_2_ and *C*_*−2*_*C*_3_ are acceptable nonlinear terms in the equation of *C*_1_. In the same way, the 3^rd^-order nonlinear term ${C}_{k}{C}_{l}{C}_{m}$ must satisfy the condition *n* = *k* + *l* + *m*. In the case of a coupling term that includes ${\dot{C}}_{l}$ and ${F}_{m}$, the same condition holds.

### Numerical simulation of the model

For numerical calculation of the force terms, Eq. (6), we use the standard Euler-Maruyama scheme for time discretization:

10 $${F}_{i}(t+{\rm{\Delta }}t)-{F}_{i}(t)=-{\kappa }_{f}{F}_{i}(t){\rm{\Delta }}t+{\kappa }_{f}{\sigma }_{i}{\xi }_{i}{({\rm{\Delta }}t)}^{1/2}.$$

To calculate Eqs (3) and (4), we use the Euler method with Δ*t* = 0.5 min. The trajectories are calculated as $x(t+{\rm{\Delta }}t)-x(t)={v}_{x}{\rm{\Delta }}t,\,y(t+{\rm{\Delta }}t)-y(t)={v}_{y}{\rm{\Delta }}t$. In the procedure for fitting, we numerically sampled *x*(*t*), *y*(*t*), and *C*_*n*_(*t*) with a time interval of 5 min, which is identical to the time interval of the image sequence in the experiment. To include observation error^13^, we add Gaussian white noise to *x*(*t*), *y*(*t*), and *C*_*n*_(*t*);

11 $$\begin{array}{c}x(t)=x(t)+{\xi }_{ox},\quad y(t)=y(t)+{\xi }_{oy},\\ \mathrm{Re}({C}_{n}(t))=\mathrm{Re}({C}_{n}(t))+{\xi }_{rn},\quad \text{Im}({C}_{n}(t))=\text{Im}({C}_{n}(t))+{\xi }_{in},\end{array}$$

where *ξ*_*i*_ are white Gaussian noises with standard deviation *σ*_0_. We estimated that the magnitude of the observation error of image processing was around 1 μm (see SI). In the fitting procedure, similar to the previous work^13^, the magnitude of the observation error *σ*_0_ was treated as a fitting parameter. We then calculate ${\boldsymbol{v}}(t)$, ${\dot{C}}_{n}(t)$ after a moving average of ${\boldsymbol{x}}(t)$ and ${C}_{n}(t)$;

12 $${\boldsymbol{v}}(t)=[{\boldsymbol{x}}(t+\tau )-{\boldsymbol{x}}(t)]/\tau ,$$

13 $${\dot{C}}_{n}(t)=[{C}_{n}(t+\tau )-{C}_{n}(t)]/\tau ,$$

with *τ* = 5 min. The properties of the trajectory are analyzed with the same procedure as that used for the experimental data.

### Fitting of the experimental data by the PRD and PRW models

In this section, we explain the method for fitting of the PRD and PRW models. Both fittings are performed at the cell-population level. For the PRD model, the number of fitting parameters is large. Thus, it is difficult to find the best fit either analytically or numerically. Thus, we manually search for a set of fitting parameters that well reproduces PDFs of velocity, deformation, phase differences (see SI), autocorrelation function of velocity and deformations, persistent length and time, rotational angle, and mean square displacement. The parameter range of manual search is shown in SI. Next, we numerically search the set of fitting parameters that locally minimize an error function *ERR* around the manually searched fitting parameters.

14 $$ERR=\sum (1-{R}_{i}^{2}),$$

where *R*_*i*_^2^ are coefficients of determination. Here we use *R*^2^ of quantile-quantile plots of experimentally-measured and numerically-calculated PDFs of velocity, deformations, and phase differences. We also include *R*^2^ of the autocorrelation functions of velocity and deformations in the error function. Since the numerical results slightly fluctuate due to the finite size of the data points, the calculated local minimum of *ERR* slightly fluctuates from time to time. Thus, we searched for the local minimum 20 times. In Table 2, we show the average and standard deviation of fitting parameters that give a local minimum of *ERR*.

For fitting by the PRW model, we fit the mean square displacement of experimental data to that of the analytical solution of the PRW model^13^ (see SI). In the fitting procedure, we numerically minimize the weighted residual sum of squares by using a nonlinear programming solver in MATLAB software.

### Calculation of persistence and turning angle

To extract the persistent motion from the trajectory, we used a time series of velocity correlation *CV*(t) with a short time interval;

15 $$CV(t)={v}_{x}(t+{\rm{\Delta }}t){v}_{x}(t)+{v}_{y}(t+{\rm{\Delta }}t){v}_{y}(t),$$

where Δ*t* = 10 min. *CV*(t) has a small value for two cases; the cell shows a rapid change in the direction of migration, or the cell almost stops. Therefore, we define that motion is persistent when *CV*(t) has a large value. For the threshold of persistent motion, a value that was 2/3 of the median of *CV*(t) was used, where the median was taken for each trajectory. In Fig. 5G, the trajectory is drawn with red lines when the cell moves persistently. We define the persistent length as the length of the red lines in Fig. 5G. We also define the rotation angle as the angle between two successive persistent trajectories^7^, shown as Δ*θ* in Fig. 5G.

### Data and materials availability

All data needed to evaluate the conclusions in the paper are present in the paper and/or the Supplementary Materials. Additional data related to this paper may be requested from the authors.

## Electronic supplementary material

Movie S1

Movie S2

Supplementary information
