# Supplementary material for: Persistent random deformation model of cells crawling on a gel surface
Source: Sci Rep. 2018 Mar 26;8:5153. doi: 10.1038/s41598-018-23540-x (PMC5980085; doi:10.1038/s41598-018-23540-x)
Supplement: Supplementary file 3 — Supplementary information [file 41598_2018_23540_MOESM3_ESM.pdf]

Supplementary Information for "Persistent random deformation model of cells crawling on a gel surface"

Hiroyuki Ebata, Aki Yamamoto, Yukie Tsuji, Saori Sasaki, Kousuke Moriyama, Thasaneeya Kuboki, Satoru Kidoaki

Laboratory of Biomedical and Biophysical Chemistry, Institute for Materials Chemistry and Engineering, Kyushu University, CE11-115, 744 Motooka, Nishi-ku, Fukuoka 819-0395, Japan

A. Movie captions

Movie S1. Time-lapse movie of fibroblasts on 35 kPa gel.

The movie corresponds to 20 h. The width of the screen corresponds to 1470 μm.

Movie S2. Movie of cell motion calculated using the PRD model.

Time evolutions of cell shape are calculated from Eqs. (2) to (7) in the main text. We used fitted parameters for 35 kPa gels listed in Table S1. To reconstruct the shape, we considered higher modes by using Eq. (S1) in this supplement. We used α_i (i = 4, 5, 6) for 35 kPa gel in Table S1. The movie corresponds to 20 h. The screen width corresponds to 1400 μm.

B. Equations for higher modes

We found that higher modes show simple relations with lower modes:

$$C_4 = \alpha_4 C_2^2, \quad C_5 = \alpha_5 C_2 C_3, \quad C_6 = \alpha_6 C_2 C_4. \tag{S1}$$

We compare the real and imaginary parts of C_n predicted from Eq. (S1) to those of actual C_n measured in the experiment. Figure S1 shows the relation between actual and predicted C_n in the same way as in Fig. 2 E in the main text. For all data, symbols follow the black line that denotes Eq. (S1). Thus, on average, higher modes C_n (n > 3) merely follow C_2 and C_3 through Eq. (S1).This result indicates that fibroblast cells have a typical shape that is defined by Eq. (S1). The estimated coefficients, α_i, are listed in Table S1. When we simulate Fig. 4 C in the main text, we use Eq. (S1) with the coefficients for 35 kPa in Table S1.

C. Relation between velocity and deformation for 120 and 410 kPa gels

For 120 and 410 kPa gels, we compare the velocity v_1 predicted from Eq. (1) in the main

text to the actual velocity $V_1 = V_x + iV_y$ measured in the experiment (Fig. S2). The bars at the lower side of the figure denote the region where 99% of the data points are found. Figure S2 shows that Eq. (1) is valid for almost all data for 120 and 410 kPa gels.

D. Akaike Information Criterion of the models

In this section, we calculate Akaike Information Criterion (AIC) for Eqs. (1) and (6) in the main text. AIC is defined as

$$AIC = 2M \log\left(2\pi \frac{\mathrm{RSS}}{M}\right) + 2k, \tag{S2}$$

where M and RSS are the number of data points and residual sum of squares, respectively. k is the number of fitting parameters. For Eq. (6), we calculate fitting parameters by numerically minimizing RSS. To minimize RSS, we use the nonlinear programming solver in MATLAB software. Generally, a smaller AIC is better than a larger one. In this work, M is much larger than k ($M \sim 10^4$, k ~ 1). Thus, RSS dominantly determines AIC. AIC values are shown in Table S2.

E. Statistical properties of velocity, deformations and trajectory for 120 kPa gel

The statistical properties of fibroblasts on 120 kPa gel are shown in Fig. S3. Black lines in the figure are fitting curves for Eqs. (2) to (7) in the main text. As with fibroblasts on 35 and 410 kPa gels, the properties of migration and deformation are well-fitted by the model.

F. Probability distribution function of phase differences

Here, we calculated the probability distribution function (PDF) of phase differences among v_1, C_n and $\dot{C}_n$, since the peaks of the PDF provide information about the correlation. Note that the phase of the velocity arg(v_1) corresponds to the direction of velocity ϕ_v, where $v_1 = v_x + iv_y$. The phases of C_n and $\dot{C}_n$ are defined as $\arg\left(C_n\right) = n\phi_n$ and $\arg\left(\dot{C}_n\right) = n\phi_{nd}$, where ϕ_n and ϕ_{nd} are the directions of deformation and extension/contraction, respectively. Due to the uniformity of the space, we only investigate phase differences that are invariant under rotational transformation, $\phi_k \to \phi_k + \theta_0$. The PDF of the phase difference $\psi_2 = \phi_2 - \phi_v$ between velocity and elongation has sharp peaks at 0°, 180° and 360° (Figs. S4 (a), (e), and (i)). These peaks show that the cells migrate along the long-axis of elongation. The PDF of the phase difference $\psi_3 = 3\phi_3 - 2\phi_2 - \phi_v$ among velocity, elongation, and triangular deformation does not have a clear peak (Figs. S4 (b), (f), and (j)). This result corresponds to no correlation between arg(v_1) and arg($C_{-2}C_3$) in Fig. 2 A in the main text. Instead, the PDFs of ψ_{2d} =

$3\phi_3$ - $2\phi_{2d}$ - ϕ_v and $\psi_{3d} = 3\phi_{3d} - 2\phi_2 - \phi_v$ have peaks (Figs. S4 (c), (d), (g), (h), (k), and (l)), which corresponds to correlations among phases in Fig. 2 B and C in the main text. All the PDFs are well-fitted by the PRD model. Thus, the PRD model successfully reproduces the correlations among phases of velocity and deformations.

G. Fitting results for the conventional PRW model

In this section, we discuss the detailed results of fitting by the conventional PRW model:

$$\dot{v}_i = -\frac{v_i}{P} + \frac{S}{\sqrt{P}} w_i, \tag{S3}$$

where $i = x, y$. P, S and w_i are persistent time, typical cell speed, and white Gaussian noise, respectively. For 2D, the mean square displacement of the PRW model[1] is

$$MSD(\Delta t) = 2S^2P^2\left(e^{-\frac{\Delta t}{P}} + \frac{\Delta t}{P} + 1\right) + 4\sigma_0^2, \tag{S4}$$

where σ_0 is measurement error. Next, we numerically minimize the weighted residual sum of squares (RSS) of MSD by using the nonlinear programming solver in MATLAB software. To prevent fitting from overemphasizing the error of MSD at a large time-lag, we multiply the residual by a weight function[1]. Here, we use $MSD(\Delta t)^{-1.3}$ as the weight function. We used S = 31.6 μm/h, P = 0.5 h, and σ_0 = 1 μm for initial value of numerical search for minimization of RSS. As shown in Figs. S5 (a) and (b), the PRW model can reproduce MSD and the autocorrelation function of velocity. However, as reported previously[1], the PRW model cannot reproduce the exponential distribution of velocity (Fig. 5 A in the main text). We cannot produce a good fit for the cumulative distribution of the persistent length (Fig. 5 H in the main text). For the experiment, the PDF of the rotational angle has a peak at 180 degrees (Fig. 5 I in the main text). However, that calculated using the PRW model has a peak at around 90 degrees. For the PRW model, we found that the peak position of the PDF shifts to a larger angle when the persistent time P decreases. However, with any P, the peak at 180 degrees cannot be reproduced by the PRW model. This misfit of the distribution of the rotational angle implies that the PRW model cannot explain the statistical properties of the direction of velocity. To address this issue, we calculate the distribution of the angular displacement $\Delta\theta(\Delta t)$ of the velocity[1]. $\Delta\theta(\Delta t)$ is defined as the angle between $\boldsymbol{v}(t)$ and $\boldsymbol{v}(t+\Delta t)$. (Fig. S5 (c) inset):

$$\Delta\theta(\Delta t) = \arccos\left(\frac{\boldsymbol{v}(t)\bullet\boldsymbol{v}(t+\Delta t)}{|\boldsymbol{v}(t)||\boldsymbol{v}(t+\Delta t)|}\right). \tag{S5}$$

As shown in Fig. S5 (d), the PDF of $\Delta\theta$ for the experimental data has peaks at 0 and 180

degrees for large Δt. However, that of the PRW model has only one peak at 0 degrees for small Δt (Fig. S5 (e)). For large Δt, the distribution of $\Delta\theta$ is flattened. On the other hand, the PRD model successfully reproduces the peak at 180 degrees for large Δt (Fig. S5 (f)). The peak at 180 degrees for large Δt should derive from reciprocal motion of the cell with a duration of a few hours (Fig. 1 C in the main text).

H. Full list of fitting parameters for the PRD model

Table S3 shows the full list of fitting parameters of Eqs. (2) to (7) for 35, 120, and 410 kPa gels. Note that β_3 and σ_6 cannot be determined separately because we do not fit C_6 in this model.

I. Parameter range of manual search of fitting parameters for the PRD model

In Table S4, we show the parameter range of initial manual search for fitting of the PRD model. To determine the range, we estimate the order of coefficients of β_1, β_2, κ_2, κ_3, κ_f, σ_2, and σ_3 from Eq. (1), half-value period of autocorrelation function of velocity and deformations, and average magnitude of deformations. We estimate the order of observation noise σ_0 from image analysis, which is explained in the next section. α_v should be positive to reproduce the PDF of phase difference between velocity and elongation[2, 3] (Fig. S4 A, E, and I). The rest coefficients of nonlinear terms should not be so large. Otherwise, the nonlinear terms cause the divergence of the numerical simulation or strong suppression of velocity and deformations. Based on above estimations, we determined the parameter range of initial manual search. Note that we did not search the entire region described in Table S4. It shows the minimum and maximum values of manual search.

J. Procedure for image analysis

We explain here the details of image analysis using MATLAB software (Fig. S6). First, we calculate the edge of a cell from the phase-contrast image by using a Sobel filter (Figs. S6 (a1) and (a2)). Since the edge of a cell is usually split up into small lines, we dilate the lines so that they connect to each other, and fill gaps in the connected line (Fig. S6 (a3)). Next, the binarized image is eroded to cancel the effect of dilation (Fig. S6 (a5)). Finally, we remove small white domains that are smaller than a threshold (Fig. S6 (a5)). We use the edges of the white domains as the shapes of the cells. As shown in Figs. S6 (b) and (c), we successfully binarize the phase-contrast image. However, thin lamellipodia and pseudopodia sometimes cannot be detected. To evaluate the measurement error of the shape and trajectory due to the procedure used for image analysis, we changed the

threshold of the Sobel filter that is dominantly affected the result of binarization. For the same cells, we calculate the change in the shape and centroid due to variation of the threshold. In the calculation, we change the threshold by 30 %. If we change the threshold much more than this, it causes a crucial error in edge detection. For the 35 kPa gel, the standard deviations of the difference in the positions of the centroid, C_2, and C_3 are 1.6, 1.0, and 0.7μm, respectively. Thus, the magnitude of the variation of the shape and centroid due to image processing is up to ~ 1 μm. This value is comparable to the measurement errors σ_0 in Table S3.

1. Wu, P.-H., Giri, A., Sun, S. X. & Wirtz, D. Three-dimensional cell migration does not follow a random walk. Proc. Natl Acad. Sci. 111, 3949–3954 (2014).
2. Hiraiwa, T., Matsuo, M. Y., Ohkuma, T., Ohta, T. & Sano, M. Dynamics of a deformable self-propelled domain. EPL 91, 20001 (2010).
3. Ohta, T. & Ohkuma, T. Deformable self-propelled particles. Phys. Rev. Lett. 102, 154101 (2009).

Designation	α_4 (μm^{-1})	α_5 (μm^{-1})	α_6 (μm^{-1})
35 kPa gel	0.032	0.067	0.033
120 kPa gel	0.027	0.058	0.029
410 kPa gel	0.026	0.054	0.029

Table S1. Parameter estimation: equations for higher modes. List of fitting parameters in Eq. (S1). The designation represents the elasticity of the gels. The fitting parameters are estimated through least-squares fitting. N = 155 for 35 kPa gel. N = 95 for 120 kPa gel. N = 119 for 410 kPa gel.

Designation	Equation 1	Equation 6
35 kPa gel	5.3522×10^4	5.2625×10^4
120 kPa gel	2.9454×10^4	2.9121×10^4
410 kPa gel	1.2952×10^4	1.2693×10^4

Table S2. Akaike Information Criterion for the models. List of AIC values for Eqs. (1) and (6). Number of data points is $M = 2.6\times10^4$ for 35 kPa gel. $M = 1.6\times10^4$ for 120 kPa gel. $M = 1.2\times10^4$ for 410 kPa gel.

	β_1 / μm^{-1}	β_2 / μm^{-1}	κ_2 / h^{-1}	κ_3 / h^{-1}	κ_f / h^{-1}	σ_2 /μm h^{-1}	σ_3 /μm h^{-1}	$\beta_3\sigma_6$ / h^{-1}
35kPa	1.21 ±0.03	0.24 ±0.01	0.38 ±0.00	1.55 ±0.05	4.16 ±0.11	5.97 ±0.00	0.98 ±0.04	1.32 ±0.03
120kPa	0.83 ±0.02	0.15 ±0.01	0.40 ±0.01	1.11 ±0.02	3.84 ±0.10	6.81 ±0.00	1.06 ±0.03	1.06 ±0.02
410kPa	0.59 ±0.02	0.07 ±0.00	0.40 ±0.01	0.51 ±0.02	4.16 ±0.11	7.17 ±0.23	1.04 ±0.04	0.85 ±0.03
	α_v / μm^{-1}	α_2 / μm^{-1}	α_3 / μm^{-1}	γ_v / μm^{-2}	γ_3 /μm^{-2} h^{-1}	σ_0 / μm		
35kPa	0.090 ±0.000	0.0079 ±0.0003	0.0040 ±0.0001	0.0032 ±0.0001	0.028 ±0.001	0.72 ±0.02		
120kPa	0.079 ±0.000	0.0060 ±0.0002	0.0054 ±0.0001	0.0027 ±0.0000	0.015 ±0.000	1.00 ±0.03		
410kPa	0.069 ±0.002	0.0131 ±0.0004	0.0049 ±0.0002	0.0018 ±0.0001	0.016 ±0.000	1.39 ±0.04		

Table S3. Full list of fitting parameters. List of fitting parameters in Eqs. (2) – (7) used in Figs. 4 and 5 [main text]. The designation represents the elasticity of the gels.

	β_1 / μm^{-1}	β_2 / μm^{-1}	κ_2 / h^{-1}	κ_3 / h^{-1}	κ_f / h^{-1}	σ_2 /μm h^{-1}	σ_3 /μm h^{-1}	$\beta_3\sigma_6$ / h^{-1}
	0.2 – 2.0	0 – 1.0	0.1– 1.0	0.1 – 2.0	2 – 5	1 – 10	0.2 – 2.0	0 – 1.5
	α_v / μm^{-1}	α_2 / μm^{-1}	α_3 / μm^{-1}	γ_v / μm^{-2}	γ_3 /μm^{-2} h^{-1}	σ_0 / μm		
	0 – 0.10	-0.02 – 0.02	-0.01 – 0.01	0 – 0.005	0 – 0.05	0.5 – 1.5		

Table S4. Range of initial manual search of fitting parameter of the PRD model. The list show the range of manual search for fitting of the PRD model.

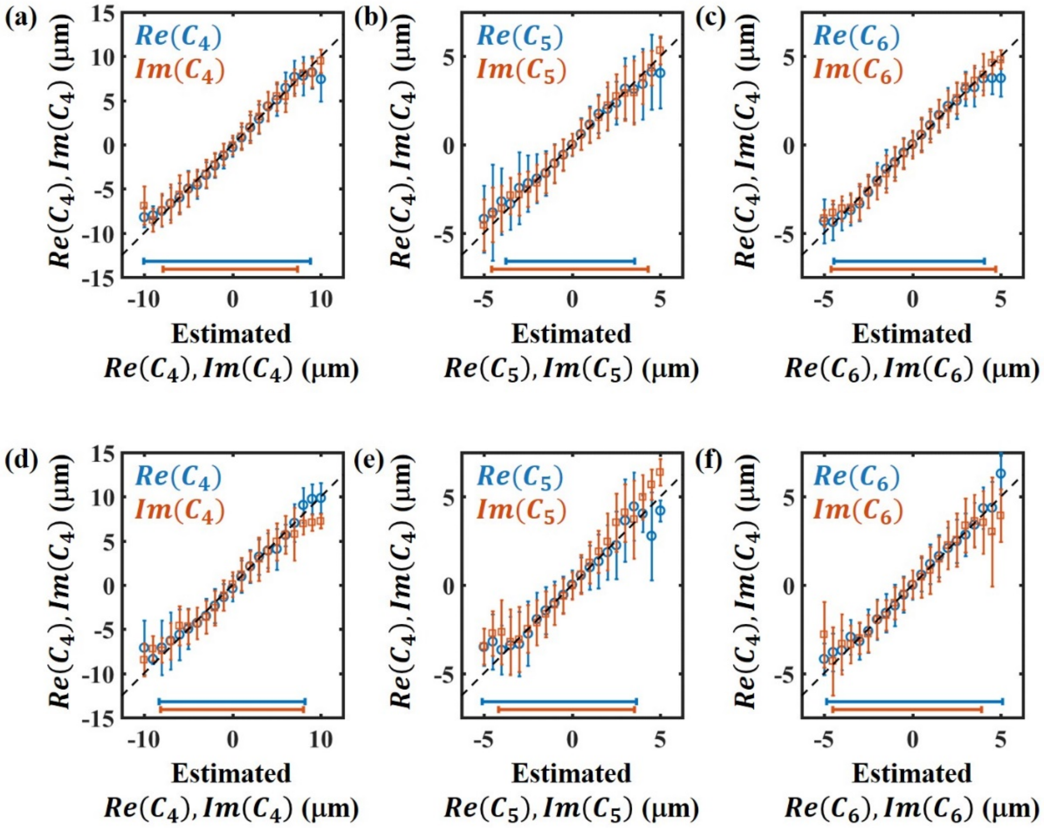

Figure S1. Relation among lower and higher modes. Relation between actual C_n and C_n estimated from Eq. (S1). Real and imaginary parts of (a, d) C_4, (b, e) C_5, and (c, f) C_6. The black dashed line indicates that the actual and estimated values are identical. Symbols denote average values. Error bar indicates the standard deviation. Blue and red bars at the lower side of the figures denote the region where 99% of the data points are found. (a – c) 120 kPa gel. (d – f) 410 kPa gel. n = 95 for 120 kPa gel. n=119 for 410 kPa gel.

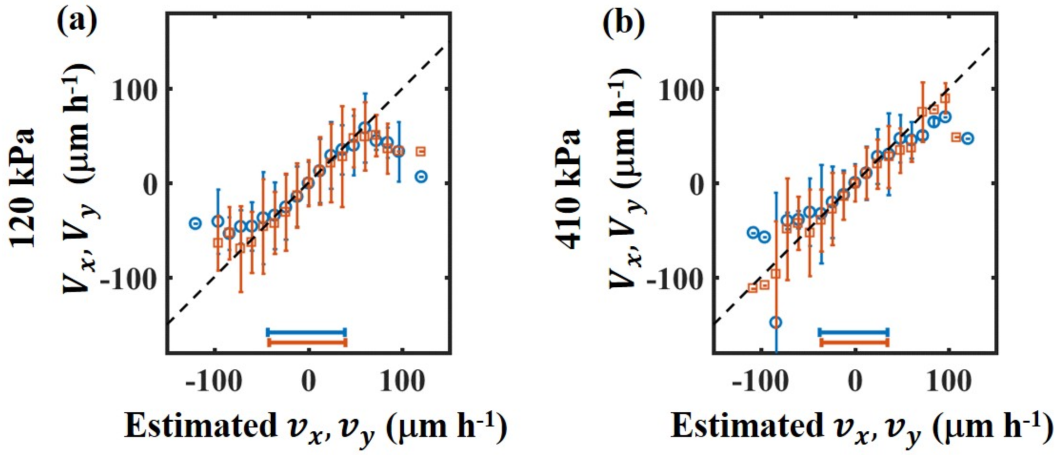

Figure S2. Relations among velocity and deformation. Relation between estimated and actual velocity (see Fig. 2 E in main text). Black dashed line represents $v_x = V_x$ ($v_y = V_y$). Symbols denote average values. Error bar indicates the standard deviation. Blue and red bars at the lower side of the figures denote the region where 99% of the data points are found. (a – d) 120 kPa gel. (e – h) 410 kPa gel. n = 95 for 120 kPa gel. n=119 for 410 kPa gel.

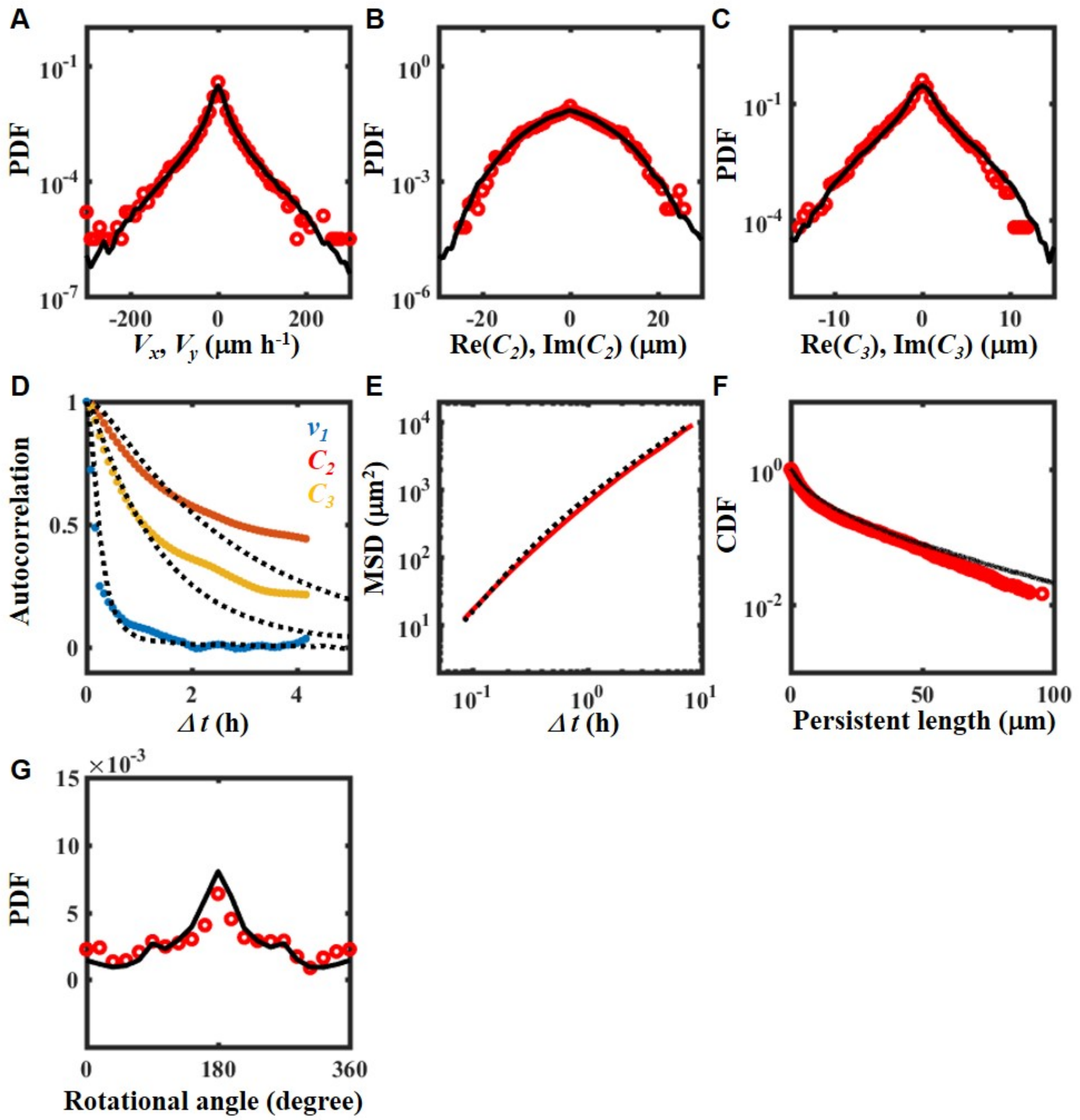

Figure S3. **Properties of cell velocity, deformation and tragectory on 120 kPa gel.** (a) – (c) Probability distribution function (PDF) of (a) velocity, (b) elongation, and (c) triangular deformation. (d) Autocorrelation functions. Red: elongation. Yellow: triangular deformation. Blue: velocity. Black dashed lines: fitted curve. (e) Mean square displacement (MSD). (f) Complementary cumulative distributions (CDF) of persistent length. (g) PDF of rotation angle. (a) – (g) black curves represent fitted curves. n = 95.

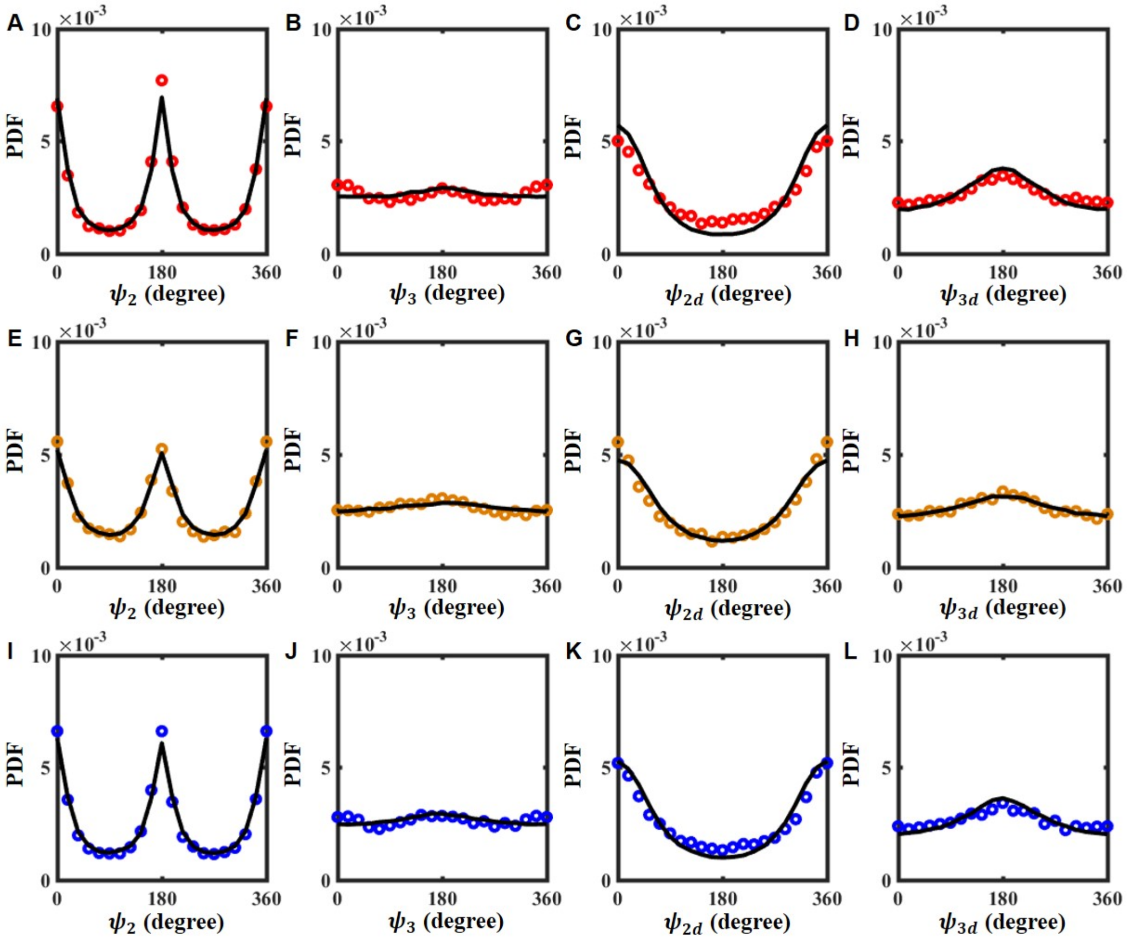

Figure S4. Probability distribution function of phase differences. See Fig. 1 G in the main text. Black curves represent fitted curves. (a), (e), (i) Probability distribution function (PDF) of ψ_2. ψ_2 is the phase difference between velocity and elongation, $\psi_2 = \phi_2 - \phi_v$. (b), (f), (j) PDF of ψ_3. ψ_3 is the phase among velocity, elongation and triangular deformation, $\psi_3 = 3\phi_3 - 2\phi_2 - \phi_v$. (c), (g), (k) PDF of ψ_{2d}. ψ_{2d} is the phase among velocity, the time derivative of elongation and triangular deformation, $\psi_{2d} = 3\phi_3 - 2\phi_{2d} - \phi_v$. (d), (h), (l) PDF of ψ_{3d}. ψ_{3d} is the phase among velocity, elongation and the time derivative of triangular deformation, $\psi_{3d} = 3\phi_{3d} - 2\phi_d - \phi_v$. (a – d) 35 kPa gel. n = 155. (e – h) 120 kPa gel. n = 95. (i – l) 410 kPa gel. n=119.

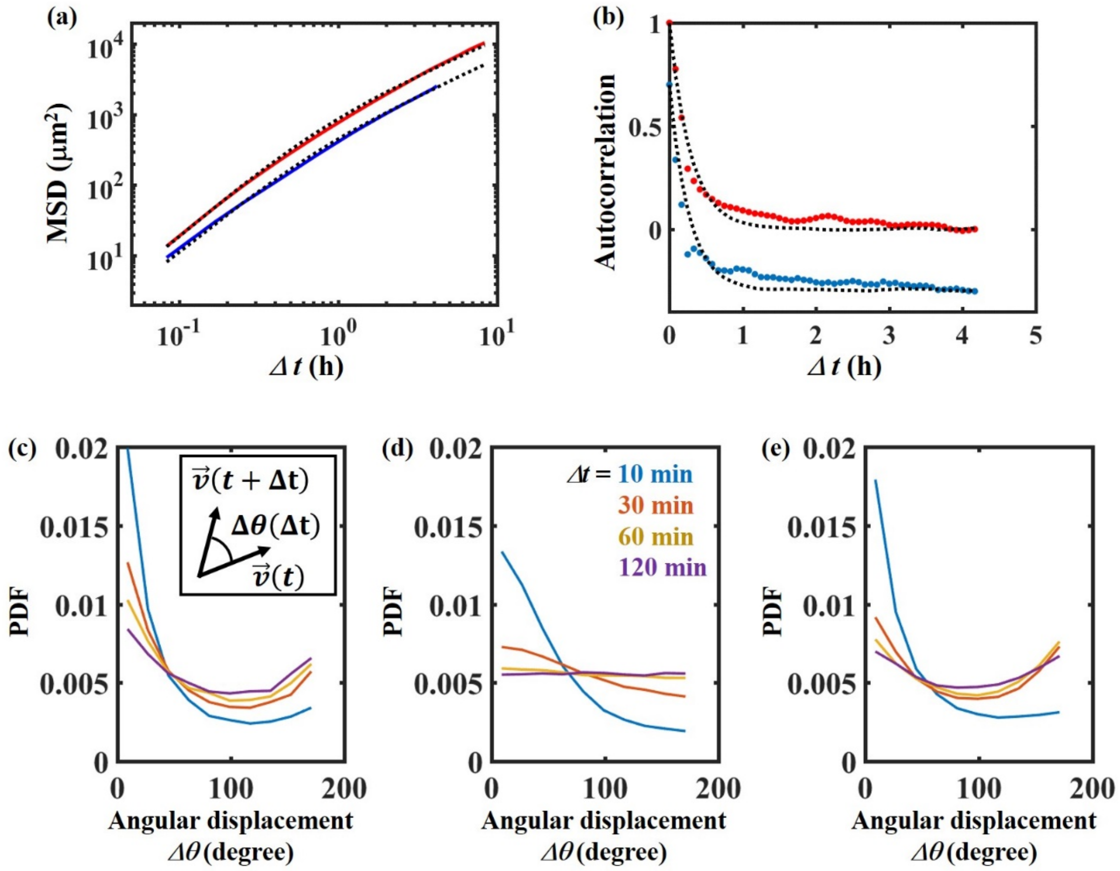

Figure S5. Fitting results by the PRW model. (a) Mean square displacement (MSD). Red: 35 kPa gel. Blue: 410 kPa gel. Black dashed lines: fitting curves with the PRW model. (b) Autocorrelation function of the velocity. Red: 35 kPa gel. Blue: 410 kPa gel. Black dashed lines: fitting curves with the PRW model. For clarity, symbols for the 410 kPa gel are shifted -0.3. (c - e) Probability distribution function of angular displacement $\Delta\theta(\Delta t)$. Blue: Δt = 10 min. Red: Δt = 30 min. Yellow: Δt = 60 min. Purple: Δt = 120 min. (c) Experimental data for the 35 kPa gel. Inset shows a schematic illustration of $\Delta\theta$. (d) Numerical results calculated from the PRW model. Parameters of the PRW model are estimated by the fitting of MSD. (e) Numerical results calculated from the PRD model. Parameters of the PRD model for the 35 kPa gel are listed in Table S3.

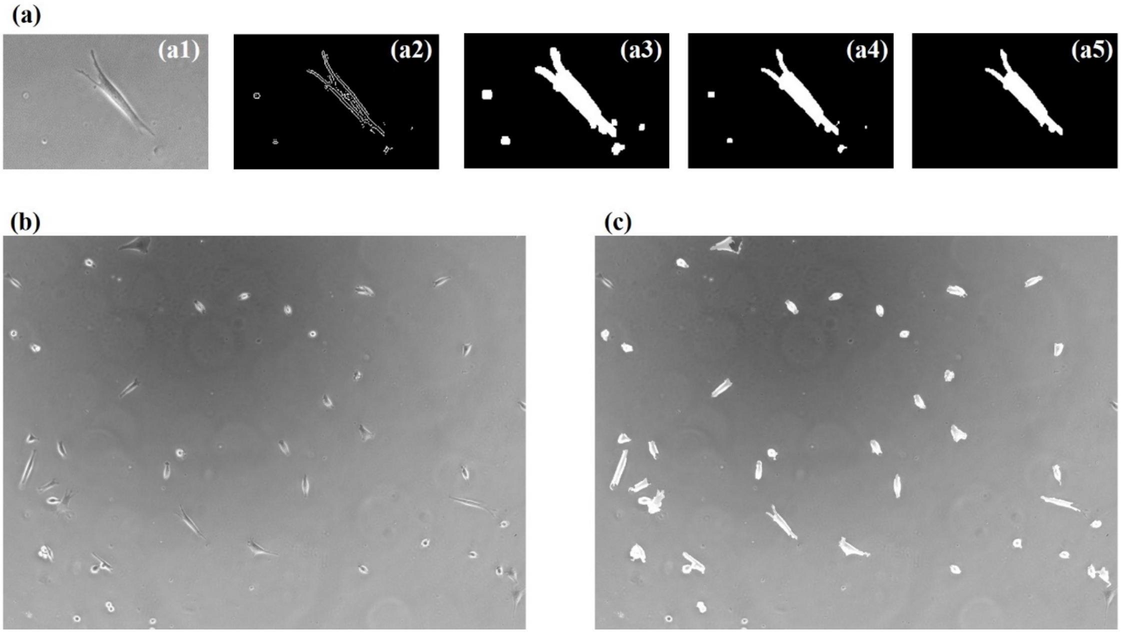

Figure S6. Procedure used for image analysis. (a) Procedure for obtaining a binarized image. (a1) Original phase-contrast image of a cell. (a2) Edge detection of a cell. (a3) We dilate the edges and fill gaps. (a4) Erosion of the binarized image. (a5) Removal of small white domains. (b) Phase-contrast image of cells. (c) Binarized image is superimposed on the phase-contrast image.
